# Cyclic Acidic Beverage Exposure Induces Formulation-Dependent Mechanical Softening and Tribological Alterations in Microhybrid and Nanohybrid Dental Resin Composites

**DOI:** 10.3390/jfb17030139

**Published:** 2026-03-11

**Authors:** Żaneta Anna Mierzejewska, Patrycja Wołosiewicz, Kamila Łukaszuk, Bartłomiej Rusztyn, Jan Borys, Bożena Antonowicz

**Affiliations:** 1 Institute of Biomedical Engineering, Faculty of Mechanical Department, Bialystok University of Technology, Wiejska 45C, 15-351 Bialystok, Poland; patrycja.wolosiewicz@gmail.com; 2 Department of Maxillofacial and Plastic Surgery, Medical University of Bialystok, M. Sklodowskiej-Curie 24A, 15-276 Bialystok, Poland; bartlomiej.rusztyn@umb.edu.pl (B.R.); jan.borys@umb.edu.pl (J.B.); 3 Department of Dental Surgery, Medical University of Bialystok, M. Sklodowskiej-Curie 24A, 15-276 Bialystok, Poland; bozena.antonowicz@umb.edu.pl

**Keywords:** dental resin composites, chemical aging, microhardness degradation, surface roughness, tribological behaviour, acidic beverages, hydrolytic degradation, wear mechanisms

## Abstract

Dental resin composites are routinely exposed to chemically aggressive beverages that may compromise long-term functional performance. This study investigated the structure–property–tribology relationships of four restorative composites (Filtek Z250, Filtek Z550, Herculite, and Herculite Ultra) subjected to cyclic immersion in beverages with different pH values. A total of 120 cylindrical specimens (7 mm diameter, 2 mm thickness; *n* = 5 per material per condition) were fabricated and exposed to mineral water, tea, coffee, Coca-Cola^®^, Cola Light^®^, and red wine for 28 days under cyclic conditions. Microhardness, surface roughness (Ra), steady-state coefficient of friction (COF), and mass variation were evaluated. All composites exhibited significant microhardness reduction after acidic exposure (*p* < 0.05), with the greatest decrease observed for Herculite Ultra in red wine (−47.4%) and Coca-Cola^®^ (−35.3%). Filtek Z250 demonstrated the highest baseline hardness and the lowest degradation susceptibility. Surface roughness changes were formulation-dependent, with Herculite Ultra showing pronounced roughening (ΔRa up to +0.074 µm), whereas Filtek Z550 exhibited erosion-driven smoothing (ΔRa down to −0.068 µm). Tribological behaviour was primarily governed by matrix softening rather than roughness alterations, with softened systems displaying unstable frictional responses (COF range: 0.127–0.697; *p* < 0.05). The results indicate that polymer matrix stability plays a more critical role in long-term functional performance than surface roughness or mass variation alone. Clinically, frequent exposure to acidic and solvent-containing beverages may accelerate mechanical and tribological degradation of susceptible composite formulations.

## 1. Introduction

Dental resin composites are widely used in restorative dentistry due to their favorable aesthetics and mechanical performance; however, their long-term durability remains strongly governed by the physicochemical conditions of the oral environment [1]. Repeated exposure to acidic beverages subjects restorations to cyclic chemical challenges that may accelerate degradation and compromise functional performance [2,3].

Acidic media promote hydrolytic degradation of dimethacrylate-based polymer networks through cleavage of ester bonds present in Bis-GMA, UDMA, and TEGDMA monomers, leading to reduced crosslink density, increased chain mobility, and matrix plasticization [4,5,6]. These molecular-scale processes directly manifest as progressive mechanical softening of the resin matrix and increased susceptibility to microstructural damage.

Simultaneously, hydrolysis of silane coupling agents at the filler–matrix interface weakens interfacial bonding and promotes filler debonding and exposure, contributing to surface roughening or erosion-dominated morphology evolution [7,8]. Consequently, chemical aging should be regarded as a coupled bulk–surface degradation process involving both polymer network deterioration and interfacial failure rather than purely superficial alteration.

Chemically induced mechanical softening and interfacial degradation strongly influence the tribological behaviour of resin composites. Reduced stiffness and compromised load transfer capacity accelerate material removal under sliding or occlusal contact, giving rise to chemically assisted wear phenomena and progressive loss of functional integrity [9,10].

Fluid sorption further contributes to degradation kinetics by facilitating hydrolysis, polymer plasticization, and elution of unreacted monomers, although its functional impact depends primarily on the chemical stability of the polymer network [11,12].

Despite extensive investigation of individual aging mechanisms, integrated understanding of how polymer network degradation and filler–matrix interfacial failure collectively govern mechanical softening, surface evolution, and tribological performance remains limited [9].

Therefore, the present study aims to establish mechanistic structure–property–tribology relationships for dental resin composites subjected to cyclic acidic exposure. By combining microhardness measurements, surface topography analysis, tribological testing, and fluid sorption analysis, this work quantitatively elucidates coupled chemical degradation pathways controlling functional deterioration.

The following null hypotheses were tested:

**(H0_1_).**  *Cyclic exposure to beverages with different pH values does not significantly affect microhardness, surface roughness, tribological behaviour, or mass variation in the investigated resin composites.*

**(H0_2_).**  *No significant differences exist among the tested composite formulations in their response to cyclic acidic exposure.*

Unlike previous studies focusing separately on microhardness, surface roughness, or wear, the present study integrates mechanical, surface, tribological, and gravimetric responses to establish a mechanistic structure–property–tribology framework. This integrated approach provides clinically relevant insight into how repeated dietary acidic challenges may compromise long-term functional stability of resin composite restorations beyond short-term surface alterations.

## 2. Materials and Methods

### 2.1. Materials

Four light-curing dental composite materials commonly used in restorative dentistry were investigated: Filtek Z250 (Z250), Filtek Z550 (Z550), Herculite (Herc), and Herculite Ultra (HercUt). The selected materials represented microhybrid and nanohybrid composite systems and were chosen due to their widespread clinical application in both anterior and posterior restorations. The main characteristics of the composites, including filler content, resin matrix composition, and manufacturer information, are summarized in Table 1.

### 2.2. Sample Preparation

A total of 120 cylindrical specimens were fabricated with a diameter of 7 mm and a thickness of 2 mm using a stainless steel mold to ensure consistent geometry and evenly distributed among 24 experimental groups (4 materials × 6 immersion media; *n* = 5 per group). Each composite material was placed into the mold in a single increment and covered with polyester strips and quartz glass slides to obtain flat, uniform surfaces and minimize oxygen inhibition. Polymerization was performed using an LED curing unit (C Woodpecker, Guilin, China) with an output intensity of approximately 1000 mW/cm^2^ for 20 s on each side of the specimen, with the curing tip positioned in direct contact with the glass surface. After removal from the mold, all samples were subjected to a standardized polishing procedure—to simulate clinical finishing—under continuous water cooling according to the manufacturers’ recommendations. Initial smoothing was performed using polishing rubber points (Kenda), followed by sequential polishing with Sof-Lex discs of decreasing abrasiveness (3M ESPE). Both upper and lower surfaces of each specimen were polished. Subsequently, specimens were stored in distilled water at 37 °C for 24 h to ensure complete post-curing prior to immersion experiments.

### 2.3. Immersion Media and Experimental Protocol

The specimens were immersed in six commonly consumed beverages representing different pH environments: mineral water (control), black tea, black coffee, Coca-Cola^®^, Cola Light^®^, and dry red wine. Carbonated beverages and wine were used directly from original containers, while tea and coffee were freshly prepared using standardized brewing conditions (2 g of tea per 100 mL of water and 6 g of coffee per 100 mL of water, brewed for 5 min at approximately 100 °C and filtered after cooling).

The pH values of all immersion media were measured using a calibrated digital pH meter (Mettler Toledo, Zurich, Switzerland) at room temperature prior to each daily immersion cycle. The mean pH values were as follows: mineral water (pH 7.64 ± 0.03), black tea (pH 5.51 ± 0.07), black coffee (pH 4.73 ± 0.05), red wine (pH 4.18 ± 0.04), Cola Light^®^ (pH 3.03 ± 0.03), and Coca-Cola^®^ (pH 2.85 ± 0.02). pH measurements were repeated daily during the aging protocol and showed no statistically significant variation.

All immersion procedures were performed after the beverages had reached room temperature. Each daily protocol consisted of four acidic exposure cycles. In each cycle, the specimen was immersed in 10 mL of the respective beverage for 10 min, followed by rinsing with distilled water for 1 min and storage in artificial saliva at 37 °C for 3 h before the next cycle. Storage in artificial saliva at 37 °C was intended to simulate intraoral conditions. After completion of the fourth cycle of the day, the specimens were kept in artificial saliva overnight until the following day. This protocol was repeated daily for 28 consecutive days.

Each specimen was assigned to a single immersion condition. After completion of the aging protocol, specimens were removed and sequentially characterized in terms of mass variation, surface roughness, microhardness, and tribological behaviour. Following tribological testing, specimens were not reused for further measurements or re-immersion. Independent specimens were used for each experimental condition.

### 2.4. Microhardness Testing

Microhardness measurements were performed using a Vickers microhardness tester (SINOWON-1, Dongguan, China) equipped with a diamond pyramidal indenter. A load of 1.96 N (200 g) was applied for a dwell time of 15 s in accordance with ISO 6507-1 standards [9]. For each specimen, five indentations were performed at randomly selected locations while avoiding overlapping impressions. Indentations were spaced apart to avoid interaction effects. The diagonal lengths of each indentation were measured using an optical microscope, and Vickers hardness values (HV) were automatically calculated by the system software. The mean hardness value for each specimen was used for statistical analysis.

### 2.5. Surface Roughness Analysis

Surface roughness measurements were performed at baseline (after polishing and 24 h storage in distilled water) and repeated after completion of the 28-day cyclic immersion protocol using the same specimens prior to tribological testing. Roughness change (ΔRa) was calculated as the difference between post-aging and baseline values according to the Equation:

(1) ∆Ra = Ra _after_ − Ra _baseline_

Thus, roughness alterations represent chemically induced surface degradation rather than wear-related modifications.

Surface topography and roughness parameters were evaluated using a confocal laser scanning microscope (Olympus LEXT OLS4000, Tokyo, Japan) operating at a wavelength of 405 nm. Three-dimensional surface reconstructions were generated from sequential optical sections acquired along the *z*-axis over a scan area of 250 × 250 µm^2^. The vertical scanning step was set at 0.1 µm. For each specimen, three randomly selected surface areas were analyzed, and the mean roughness values were calculated. Both linear and areal roughness parameters were obtained, with particular focus on the arithmetic mean roughness (Ra) and surface roughness (Sa) as indicators of surface degradation. Surface roughness measurements were conducted prior to tribological testing to avoid wear-induced surface alteration. Representative two-dimensional micrographs and three-dimensional surface maps were recorded to visualize morphological changes following immersion in different beverages.

### 2.6. Tribological Testing

Tribological behaviour of the composite specimens was evaluated using a pin-on-disc tribometer operating under wet conditions to simulate the oral environment. Prior to testing, specimens were immersed in their respective beverages for the designated exposure period and gently rinsed with distilled water to remove residual fluid. Ceramic spherical counterbody with a diameter of 6 mm was employed to ensure reproducible contact conditions and minimize variability associated with natural enamel antagonists, while providing chemically stable and wear-resistant contact surfaces under wet testing conditions.

A normal load of 5 N was applied to reproduce contact stresses representative of posterior masticatory conditions. Considering the spherical counterbody geometry, the corresponding Hertzian contact pressures were estimated to range between approximately 50 and 150 MPa, consistent with values reported for occlusal contacts during normal mastication. Previous studies have demonstrated that contact loads between 1 and 10 N adequately simulate intraoral sliding and wear processes of restorative materials under wet conditions [9,10]. The sliding speed was set to 0.05 m/s. All experiments were performed at room temperature in the presence of artificial saliva to maintain consistent wet conditions throughout the tests.

The coefficient of friction (COF) was continuously recorded as a function of sliding distance. Steady-state COF values were determined from the stabilized friction regime corresponding to the final 30% of the total sliding distance to exclude initial running-in effects.

Tests were conducted in triplicate, and mean values were used for statistical analysis. In the present study, quantitative wear volume or wear depth measurements were not determined. Wear behaviour was assessed qualitatively based on post-test surface observations and frictional response characteristics. Therefore, interpretations regarding wear severity are based on frictional stability and morphological evaluation rather than volumetric material loss quantification.

### 2.7. Mass Variation Analysis

Mass variation in the composite specimens was assessed at baseline and after completion of the aging protocol using a gravimetric method. Each specimen was gently blotted with absorbent paper to remove residual surface liquid, blot-dried for 30 s, and weighed within 60 s using an analytical balance (Mettler Toledo, accuracy ±0.001 g). Each specimen was weighed three times and the mean value was used for analysis.

The percentage mass variation (Δm%) was calculated according to the following Equation:

(2) $$\Delta m(\%)=\frac{m_{28}-\;m_{0}}{m_{0}}\;\times \;100$$

where $m_{0}$ represents the initial mass before aging and $m_{28}$ represents the mass after 28 days of cyclic exposure. Prior to weighing, specimens were gently blotted dry with absorbent paper to remove superficial liquid. No desiccator drying was performed, as the objective of the present study was not to determine standardized water sorption or solubility, but rather to assess net mass variation under cyclic chemical exposure conditions. Therefore, the absorbed fluid fraction within the polymer matrix was intentionally preserved to reflect the chemically aged state of the material.

### 2.8. Statistical Analysis

Statistical analysis was performed using Statistica software. Data normality was assessed using the Shapiro–Wilk test, and homogeneity of variances was evaluated using Levene’s test. Depending on data distribution, one-way analysis of variance (ANOVA), ANOVA with Welch correction, or the nonparametric Kruskal–Wallis test was applied to determine the effect of immersion media on all measured parameters, including microhardness, surface roughness, coefficient of friction, wear behaviour, and fluid sorption. Post hoc comparisons were conducted using Tukey’s test for parametric data or Dunn’s test for nonparametric data. Correlation analysis between selected parameters was performed using Pearson or Spearman correlation coefficients as appropriate. The level of statistical significance was set at α = 0.05. An a priori sample size calculation was performed using G*Power 3.1 software (Heinrich Heine University Düsseldorf, Düsseldorf, Germany) for a two-way ANOVA model (4 × 6 design). Assuming a large effect size (f = 0.40), α = 0.05, and power = 0.80, the minimum required sample size was calculated as *n* = 5 per subgroup. Microhardness values after 28 days were analyzed using two-way ANOVA with material (4 levels) and immersion medium (6 levels) as fixed factors. Interaction effects between factors were also evaluated. The level of statistical significance was set at α = 0.05. Surface roughness values after 28 days (Ra^2^) were analyzed using one-way ANOVA separately for each composite material to evaluate the effect of immersion medium (*n* = 5 per group). Statistical significance was set at α = 0.05. Post hoc comparisons were performed using Tukey’s test.

## 3. Results

### 3.1. Chemically Induced Mechanical Softening of Dental Resin Composites

Two-way ANOVA demonstrated a significant main effect of material (F(3,96) = 578.31, *p* < 0.001) and immersion medium (F(5,96) = 14.35, *p* < 0.001) on microhardness after 28 days. A statistically significant interaction between material and immersion medium was also observed (F(15,96) = 2.47, *p* = 0.004), indicating formulation-dependent differences in degradation response. Statistical analysis revealed a significant effect of immersion medium on microhardness for all investigated composites (*p* < 0.001). All investigated composite materials exhibited progressive chemically induced mechanical softening following cyclic immersion in beverages with different pH values. The extent of microhardness reduction was strongly dependent on both the chemical aggressiveness of the immersion medium and the composite formulation. Baseline microhardness values and values obtained after 28 days of aging are summarized in Table 2, while the corresponding percentage reductions relative to baseline are presented in Table 3.

Herculite exhibited intermediate degradation resistance compared with Herculite Ultra. The greatest microhardness losses occurred after exposure to mineral water and red wine (approximately 33%), whereas immersion in Coca-Cola^®^ resulted in comparatively lower softening (25.3%), as summarized in Table 3. Tea and coffee induced moderate mechanical degradation.

Herculite Ultra showed the lowest initial microhardness and the highest susceptibility to chemical degradation. As shown in Table 3, microhardness decreased most markedly in red wine (47.4% reduction) and Coca-Cola^®^ (35.3% reduction), indicating pronounced resin matrix softening under strongly acidic conditions. Moderate but statistically significant reductions were also observed following immersion in coffee and Cola Light^®^.

Filtek Z250 demonstrated the highest baseline hardness and the greatest resistance to chemically induced softening. Although significant microhardness reductions were observed in all media, the overall magnitude of degradation remained lower than that recorded for Herculite-based composites. According to Table 3, the largest decrease was detected following exposure to Cola Light^®^ (27.3%), while red wine produced the lowest reduction (21.6%).

Similarly, Filtek Z550 exhibited progressive hardness loss, with the most pronounced degradation observed after immersion in Coca-Cola^®^ (34.7%). Exposure to Cola Light^®^ and red wine resulted in moderate reductions, whereas mineral water, tea, and coffee produced less severe softening effects.

Statistical analysis confirmed a significant influence of immersion medium on microhardness for all composite systems (*p* < 0.05). One-way ANOVA revealed highly significant differences among beverages for Herculite Ultra (*p* < 0.001) and Filtek Z550 (*p* = 0.005), while Welch-corrected ANOVA indicated significant effects for Herculite (*p* = 0.002). For Filtek Z250, the Kruskal–Wallis test also demonstrated statistically significant differences among immersion media (*p* = 0.034). Post hoc comparisons identified red wine and Coca-Cola^®^ as the primary drivers of mechanical softening, particularly for Herculite-based composites.

Overall, chemically aggressive media induced substantial resin matrix softening across all materials, with distinct susceptibility patterns among composite systems, highlighting the critical role of composite formulation in resistance to acid-mediated degradation.

### 3.2. Surface Morphology Evolution and Roughness Alterations After Chemical Degradation

One-way ANOVA revealed a statistically significant effect of immersion medium on surface roughness after 28 days (Ra^2^) for all investigated composites: Herculite Ultra (*p* < 0.001), Herculite (*p* = 0.001), Filtek Z250 (*p* = 0.004), and Filtek Z550 (*p* < 0.001). Post hoc analysis identified significant differences between selected immersion media within each composite group. Table 3 summarizes the baseline surface roughness (Ra) values of the investigated composite materials together with the corresponding values measured after 28 days of cyclic immersion and the calculated roughness changes (ΔRa). The results demonstrate that chemically induced surface evolution was strongly dependent on composite formulation, with both pronounced surface roughening and erosion-driven smoothing phenomena observed.

**Figure 1:**
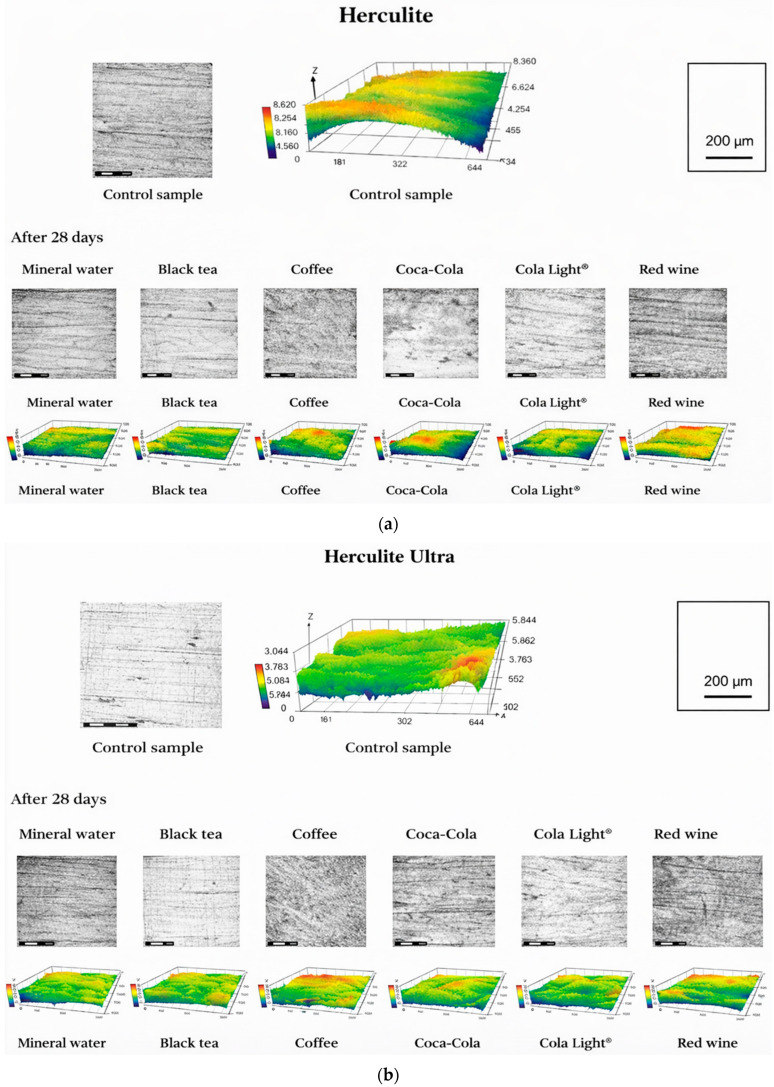
Representative 2D confocal micrographs and corresponding 3D surface topography maps of (**a**) Herculite, (**b**) Herculite Ultra condition and after 28 days of immersion in mineral water, black tea, coffee, Coca-Cola^®^, Cola Light^®^, and red wine, illustrating beverage-dependent changes in surface roughness.

For Herculite, surface roughness alterations were comparatively limited and strongly medium-dependent. Mineral water and tea produced moderate roughness increases, whereas coffee and Coca-Cola^®^ resulted in negligible changes relative to baseline. Notably, Cola Light^®^ induced slight surface smoothing (ΔRa = −0.010 µm), suggesting partial removal of surface asperities. Red wine caused minimal morphological alteration. These contrasting responses are visualized in Figure 1a.

Herculite Ultra exhibited consistent surface roughening across all immersion media. The most pronounced increase in Ra occurred following exposure to coffee (ΔRa = +0.074 µm), while Coca-Cola^®^ and Cola Light^®^ also induced substantial roughness growth. Moderate but systematic increases were observed after immersion in mineral water, tea, and red wine, indicating progressive resin matrix degradation and filler–matrix interface deterioration. Representative two-dimensional micrographs and three-dimensional surface maps illustrating these roughness changes are presented in Figure 1b.

**Figure 2:**
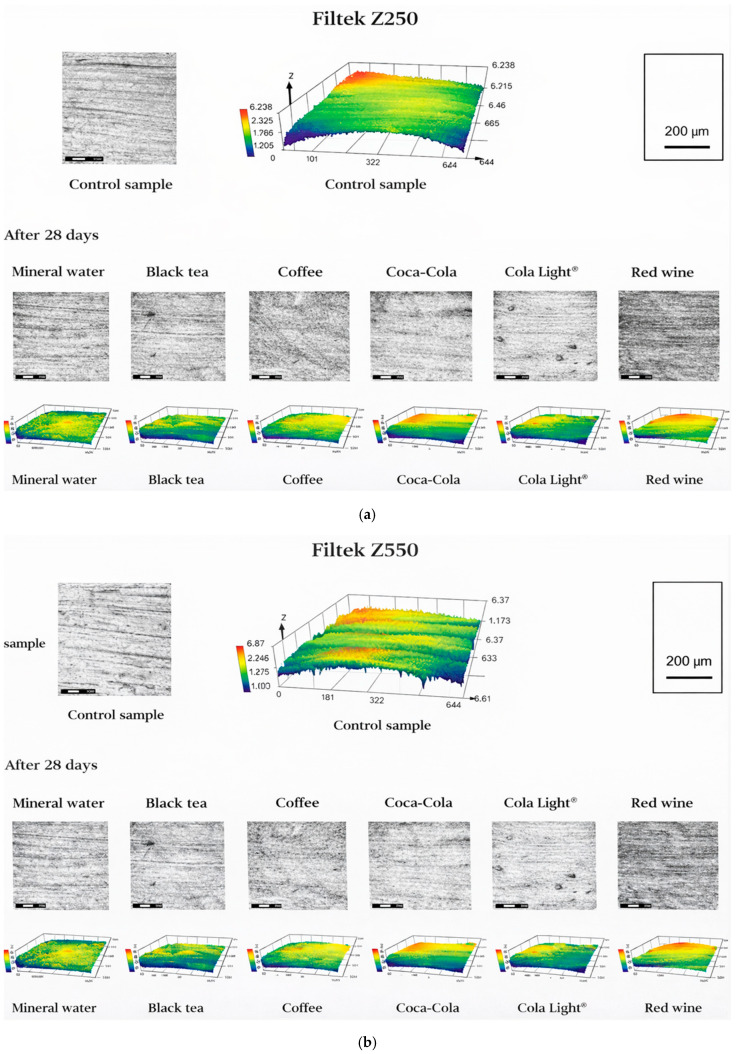
Representative 2D confocal micrographs and corresponding 3D surface topography maps of (**a**) Filtek Z250, (**b**) Filtek Z550 condition and after 28 days of immersion in mineral water, black tea, coffee, Coca-Cola^®^, Cola Light^®^, and red wine, illustrating beverage-dependent changes in surface roughness.

Filtek Z250 showed substantial surface roughening following exposure to all beverages. The largest Ra increase was observed in Cola Light^®^ (ΔRa = +0.090 µm), followed by red wine and coffee, which also caused pronounced deterioration. Even mineral water induced noticeable roughness growth, highlighting the susceptibility of this composite to prolonged fluid interaction. Representative degraded surface morphologies are shown in Figure 2a.

Conversely, Filtek Z550 demonstrated a consistent tendency toward surface smoothing rather than roughening. All immersion media resulted in negative ΔRa values, with the greatest reduction observed after exposure to Coca-Cola^®^ (ΔRa = −0.068 µm). Tea, coffee, Cola Light^®^, and red wine also produced moderate smoothing effects. This behaviour suggests erosion of the superficial resin layer and removal of loosely bound filler particles, leading to smoother but chemically compromised surfaces, as illustrated in Figure 2b.

Collectively, the roughness results reveal distinct surface degradation mechanisms among the investigated composites. Herculite Ultra and Filtek Z250 primarily underwent degradation-driven surface roughening associated with resin matrix dissolution and filler exposure, whereas Filtek Z550 exhibited erosion-dominated surface smoothing. These contrasting morphological responses underscore the critical role of composite microstructure and resin composition in controlling surface stability under chemically aggressive conditions.

### 3.3. Influence of Chemical Degradation on Tribological Behaviour and Wear Resistance

Tribological testing revealed pronounced formulation-dependent differences in frictional behaviour and wear resistance following chemically induced degradation. The influence of acidic exposure on steady-state coefficients of friction (COF) was strongly dependent on the dominant surface degradation mechanism, with both friction enhancement and erosion-driven smoothing effects observed (Table 4).

Herculite showed moderate tribological stability with mixed frictional responses. Acidic beverages such as tea, coffee, and Coca-Cola^®^ increased COF values relative to baseline, whereas exposure to Cola Light^®^ and red wine resulted in pronounced friction reduction, consistent with partial surface smoothing. Wear track analysis revealed shallow grooves and limited material loss compared with Herculite Ultra, indicating improved resistance to degradation-driven wear.

Herculite Ultra exhibited the highest susceptibility to tribological deterioration. Although exposure to tea resulted in increased COF values relative to baseline, immersion in strongly acidic media such as Coca-Cola^®^ and Cola Light^®^ led to substantial friction reduction, reaching a minimum COF of 0.127 after Cola Light^®^ exposure. These reductions were accompanied by unstable friction curves and pronounced wear track morphology suggesting greater surface degradation, indicating severe resin matrix erosion and material removal. Mineral water and tea produced comparatively higher friction levels, although wear depth remained greater than for other composite systems.

Filtek Z250 demonstrated enhanced wear resistance relative to Herculite-based composites. Although exposure to tea and Coca-Cola^®^ produced increased COF values, immersion in other media resulted in moderate friction changes. Wear tracks remained relatively smooth with limited material removal, reflecting greater mechanical stability.

Conversely, Filtek Z550 exhibited the most stable tribological performance. While Cola Light^®^ induced a slight COF increase and Coca-Cola^®^ produced minimal friction change, most immersion media led to friction reduction relative to baseline. Shallow and uniform wear tracks confirmed minimal material loss despite prolonged chemical exposure.

Tribological behaviour was strongly governed by chemically induced changes in mechanical properties and surface morphology. Composites undergoing pronounced matrix softening and surface roughening, particularly Herculite Ultra, exhibited unstable frictional responses and increased susceptibility to surface degradation under sliding. In contrast, materials characterized by higher hardness retention and erosion-driven surface smoothing, such as Filtek Z550, demonstrated superior tribological stability.

### 3.4. Mass Variation Associated with Hydrolytic Degradation

Gravimetric analysis revealed distinct formulation-dependent mass variation in the composite specimens after 28 days of cyclic aging in different immersion media (Table 5). The observed mass changes reflect the combined effects of fluid uptake within the composite structure and material loss associated with resin matrix degradation and filler–matrix interfacial deterioration during chemical aging.

Positive mass variation values indicate net mass gain dominated by fluid penetration into the polymer network, whereas negative values suggest prevailing material loss resulting from matrix dissolution and interfacial debonding processes. The balance between these competing mechanisms was strongly dependent on composite formulation and immersion medium.

Herculite Ultra exhibited generally limited mass variation across most immersion conditions. The highest positive mass change was observed after exposure to Cola Light^®^ (+2.931%), indicating enhanced fluid penetration under chemically aggressive conditions. In contrast, negative mass variation recorded after immersion in coffee (−1.213%) and red wine (−0.707%) suggests localized material loss exceeding fluid uptake, consistent with pronounced matrix degradation phenomena.

Herculite demonstrated the highest overall susceptibility to mass variation among the investigated composites. Substantial positive mass changes were recorded after exposure to Coca-Cola^®^ (+4.716%) and coffee (+4.234%), indicating significant fluid penetration into the composite structure. Negative mass variation was observed only after Cola Light^®^ exposure (−1.715%), suggesting a shift toward degradation-dominated material loss under strongly acidic conditions.

Filtek Z250 exhibited comparatively stable mass behaviour, with limited positive mass variation in mineral water (+1.914%) and cola-based beverages (+0.622–1.340%). Slight negative mass changes observed after immersion in tea (−0.048%), coffee (−0.574%), and red wine (−2.440%) indicate a predominance of material loss over fluid uptake in these media, particularly for red wine.

Similarly, Filtek Z550 showed moderate mass variation, with the highest positive mass change observed after exposure to Cola Light^®^ (+2.899%) and Coca-Cola^®^ (+1.628%). Red wine immersion resulted in near-neutral mass variation (−0.203%), suggesting a balance between fluid penetration and material dissolution processes.

Overall, Herculite-based composites exhibited greater susceptibility to mass variation associated with chemical aging, whereas Filtek-based systems demonstrated enhanced resistance to both fluid penetration and degradation-driven material loss. These results highlight the critical influence of resin matrix composition and filler–matrix interfacial stability on the chemical durability of dental resin composites under cyclic acidic exposure.

### 3.5. Structure–Property–Tribology Relationships Governing Functional Degradation

**Figure 3:**
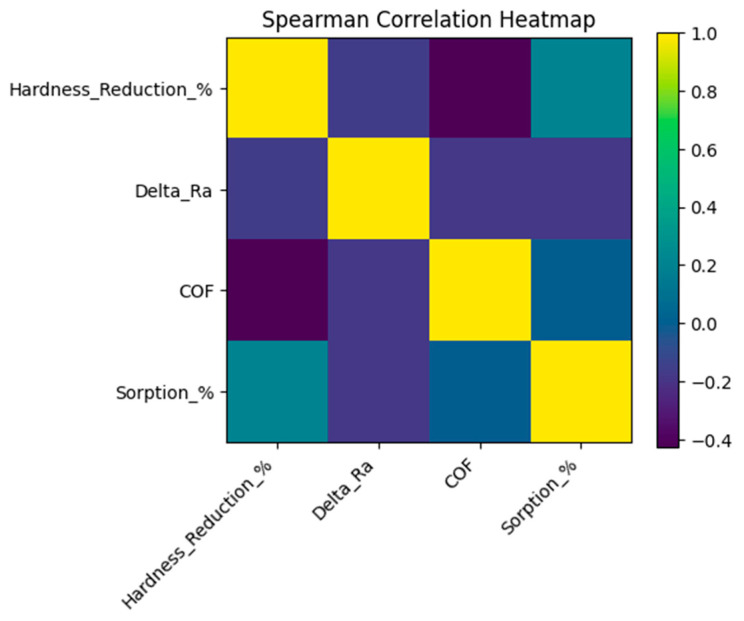
Spearman correlation heatmap illustrating relationships between microhardness reduction, surface roughness changes (ΔRa), mass variation associated with chemical aging, and steady-state coefficient of friction (COF) for all composite systems after cyclic acidic exposure. Positive correlations are shown in warm colors, while negative correlations are indicated in cool colors.

Correlation analysis based on averaged experimental values revealed clear mechanistic interdependencies between chemically induced mechanical softening, surface morphology evolution, mass variation associated with chemical aging, and tribological performance of the investigated composite materials (Figure 3).

A moderate negative correlation was observed between microhardness reduction and steady-state coefficient of friction (Spearman r = −0.43), indicating that progressive resin matrix degradation and mechanical softening promoted friction reduction rather than friction enhancement. Composites exhibiting the greatest hardness loss, particularly Herculite Ultra, showed unstable frictional responses associated with erosion-driven material removal and surface transformation.

Surface roughness changes exhibited only weak correlation with frictional behaviour (r = −0.18), suggesting that topographical alterations alone were not the dominant factor governing tribological response. Instead, the mechanical integrity of the polymer matrix appears to play a more critical role in controlling frictional stability following chemical degradation.

Mass variation demonstrated limited correlation with both microhardness reduction (r = 0.20) and friction coefficients (r = −0.01), indicating that the extent of mass change alone did not directly predict functional deterioration severity. These results further support that chemical susceptibility of the polymer network, rather than total mass variation, governs degradation-induced mechanical weakening and tribological performance.

Taken together, the correlation analysis supports a coupled degradation mechanism in which chemically induced matrix softening initiates surface morphology transformation through filler–matrix debonding and erosion processes, ultimately controlling frictional behaviour and wear resistance.

## 4. Discussion

The cyclic immersion protocol applied in this study was designed to simulate repeated short-term acidic challenges associated with daily beverage consumption rather than continuous chemical exposure. This approach reflects clinically relevant dietary conditions and has been widely employed to reproduce chemical degradation of resin-based composites in vitro [1,13,14,15].

The substantial microhardness reduction observed for all composite systems confirms acid-catalyzed hydrolysis of ester bonds within dimethacrylate-based polymer networks as the dominant degradation mechanism [1,4,16]. Similar hydrolytic degradation pathways have been widely described for methacrylate-based composites exposed to aqueous or acidic environments [5,8]. Cleavage of these bonds reduces crosslink density, increases chain mobility, and promotes polymer plasticization, directly manifesting as mechanical softening of the resin matrix. However, degradation severity was not governed solely by beverage pH. Media containing organic acids and ethanol, particularly red wine, produced degradation effects comparable to or exceeding those induced by strongly acidic carbonated drinks, demonstrating that solvent–polymer interactions and chemical composition critically modulate hydrolytic susceptibility [17,18].

Interestingly, water immersion resulted in microhardness reduction comparable to or exceeding that observed in certain acidic media. This finding may be attributed to water-induced plasticization and hydrolytic degradation of the dimethacrylate network. Water molecules diffuse into the polymer matrix, reduce intermolecular interactions, and increase chain mobility, thereby lowering crosslink density and promoting bulk softening [11,12,15]. Unlike acidic solutions, which may predominantly induce surface erosion, water can penetrate more deeply into the material and affect subsurface mechanical integrity. Consequently, microhardness reduction does not necessarily correlate directly with beverage acidity.

Formulation-dependent degradation responses further highlight the central role of resin matrix chemistry and filler–matrix interfacial stability. The greater susceptibility of Herculite-based composites suggests lower chemical resistance of their polymer networks and weaker interfacial bonding, whereas Filtek systems exhibited enhanced stability consistent with more robust resin architectures and improved silane coupling efficiency [4,7,19,20].

The distinct degradation patterns observed among the investigated materials can be rationalized by differences in resin matrix composition and filler architecture. Herculite Ultra contains PEGDMA, a relatively hydrophilic monomer that may increase water uptake and facilitate hydrolytic ester bond cleavage compared with more hydrophobic Bis-EMA-containing systems such as Filtek Z250 [12,21]. Increased hydrophilicity enhances diffusion of acidic media into the polymer network, accelerating plasticization and crosslink density reduction.

Furthermore, differences in filler loading and particle size distribution between microhybrid and nanohybrid composites may influence interfacial stability. Nanohybrid systems such as Filtek Z550 contain a higher proportion of nanoscale fillers with larger interfacial surface area, potentially promoting more homogeneous stress distribution and improved silane coupling efficiency. In contrast, microhybrid systems may exhibit more pronounced filler debonding once interfacial hydrolysis is initiated [22,23].

Importantly, degradation severity did not correlate strictly with beverage pH. Red wine produced substantial softening despite moderate acidity, likely due to the combined action of organic acids and ethanol, which can enhance polymer swelling and solvent–polymer interactions. This observation confirms that chemical composition of immersion media, rather than pH alone, governs degradation kinetics.

Surface morphology evolution reflected microstructural consequences of polymer degradation and interfacial failure. The pronounced roughening observed for Herculite Ultra and Filtek Z250 is consistent with preferential resin matrix dissolution followed by filler exposure and debonding processes [17,19]. In contrast, erosion-driven surface smoothing detected for Filtek Z550 indicates removal of degraded superficial resin layers and loosely bound filler particles, resulting in smoother but chemically compromised surfaces [18]. These contrasting degradation modes demonstrate that surface roughness alterations may arise from fundamentally different microstructural pathways depending on composite formulation.

Tribological behaviour was governed primarily by chemically induced mechanical softening rather than surface roughness changes alone. Composites undergoing severe matrix degradation transitioned toward chemically assisted wear regimes characterized by polymer softening, adhesive material transfer, and accelerated erosion-driven material removal, particularly evident for Herculite Ultra [10,23]. Increased roughness in these softened systems further promoted abrasive interactions, amplifying wear severity [20,22]. In contrast, composites retaining greater mechanical integrity, especially Filtek Z550, operated within mild wear regimes dominated by limited adhesive and erosive mechanisms, resulting in stable frictional response and minimal material loss [10,18]. These findings confirm that chemically assisted wear mechanisms dominate over purely abrasive processes under acidic aging conditions, with polymer network softening acting as the primary trigger for accelerated material removal. Surface roughness variations therefore represent a consequence of underlying chemical degradation rather than an independent driver of tribological performance [24].

The mass variation results should be interpreted within the context of the applied methodology. As specimens were not desiccator-dried prior to weighing, the measured mass changes represent the combined effect of fluid uptake and material loss under simulated exposure conditions rather than standardized sorption values according to ISO protocols. Future studies may incorporate controlled desiccation procedures to isolate sorption-specific mechanisms. Mass variation associated with chemical aging acted as a degradation enabler rather than the primary controlling factor. The measured mass changes reflect the combined effects of fluid penetration into the polymer network and material loss resulting from resin matrix dissolution and filler–matrix interfacial deterioration. Although liquid uptake facilitates hydrolysis and polymer plasticization [11,12,25,26], the chemical stability of the polymer network ultimately governs the extent to which mass variation translates into mechanical weakening and tribological deterioration.

**Figure 4:**
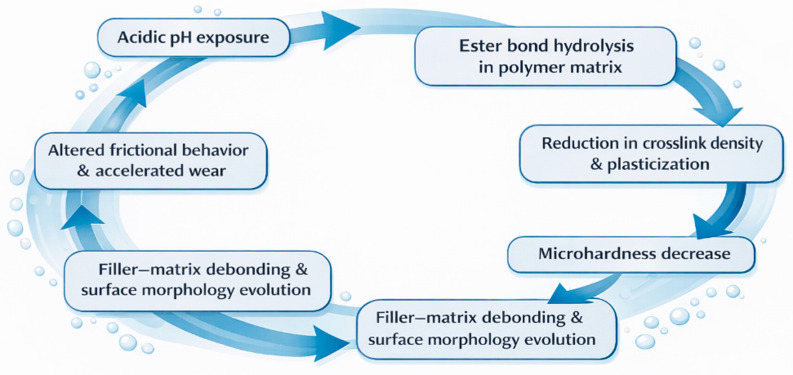
Conceptual degradation pathway of dental resin composites under acidic exposure.

The coupled degradation mechanisms identified in this study are schematically summarized in Figure 4. As illustrated, cyclic exposure to chemically aggressive media initiates polymer network hydrolysis, leading to progressive matrix softening and interfacial failure. These molecular- and microstructural-scale processes drive surface morphology transformation through filler exposure or erosion-driven smoothing and promote transition toward chemically assisted wear regimes under sliding contact. This integrated structure–property–tribology pathway explains the formulation-dependent functional degradation observed among microhybrid and nanohybrid composite systems [27,28,29].

From a clinical perspective, these degradation mechanisms have direct implications for restoration longevity. Roughening-dominated degradation promotes bacterial adhesion and staining, while erosion-driven softening compromises wear resistance and marginal integrity [1,14,30]. Frequent consumption of acidic and solvent-containing beverages may therefore accelerate functional deterioration of composite restorations, particularly for materials with lower chemical stability.

Several limitations should be acknowledged. The in vitro aging protocol represents an accelerated chemical exposure model and does not incorporate mechanical fatigue, thermal cycling, or enzymatic activity present in the oral environment. Nevertheless, controlled cyclic exposure enables isolation of chemical degradation mechanisms and provides valuable comparative insight into formulation-dependent susceptibility.

The absence of quantitative wear volume or depth measurements represents a limitation of the present study. Future investigations incorporating profilometric or 3D volumetric wear analysis would allow more precise quantification of degradation-driven material loss.

Overall, the present results demonstrate that chemically induced degradation of dental resin composites is governed by tightly coupled polymer network hydrolysis, filler–matrix interfacial failure, and mechanically assisted surface transformation rather than isolated property changes. Progressive matrix softening driven by ester bond cleavage represents the primary degradation trigger controlling subsequent surface evolution and tribological deterioration, in agreement with chemically assisted wear concepts reported for polymeric systems [31,32].

## 5. Conclusions

Cyclic exposure to acidic beverages induces coupled polymer matrix softening and filler–matrix interfacial degradation in dental resin composites, resulting in formulation-dependent mechanical and tribological responses.

Mechanical integrity of the resin matrix appears to be a more critical determinant of functional stability than surface roughness or overall mass variation alone.

Herculite-based composites showed greater susceptibility to chemical degradation, whereas Filtek-based systems demonstrated comparatively higher resistance, highlighting the importance of resin chemistry and interfacial stability in predicting long-term performance under acidic oral conditions.

## Figures and Tables

**Table 1:** Characteristics of investigated dental composite materials.

Composite (Abbreviation)	Type	Filler	Filler Content (wt%)	Resin Matrix Composition	Declared Hardness (HV)	Manuf.
Herculite (Herc)	Microhybrid	BaAlSiO_4_, SiO_2_	78	Bis-GMA, UDMA, TEGDMA	60–85	Kerr, Orange, CA, USA
Herculite Ultra (HercUt)	Nanohybrid	BaAlSiO_4_, SiO_2_	79	Bis-GMA, UDMA, TEGDMA, PEGDMA	80–100	
Filtek Z250 (Z250)	Microhybrid	zirconia/silica	82	Bis-GMA, UDMA, Bis-EMA	75–90	3M ESPE, St. Paul, MN, USA
Filtek Z550 (Z550)	Nanohybrid	zirconia/silica nanoparticles	82	Bis-GMA	80–95	

**Table 2:** Vickers microhardness at baseline and after 28 days of immersion (HV).

Composite	Baseline (Mean ± SD)	Water	Tea	Coffee	Coca-Cola^®^	Cola Light^®^	Red Wine
Herculite	76.59 ± 3.43	51.30 ^a^	52.84 ^ab^	54.64 ^b^	57.20 ^b^	55.28 ^b^	50.88 ^a^
Herculite Ultra	65.67 ± 3.04	42.82 ^a^	45.66 ^ab^	47.12 ^b^	42.52 ^a^	44.50 ^ab^	34.56 ^c^
Filtek Z250	89.79 ± 4.14	68.52 ^a^	67.28 ^a^	69.92 ^a^	66.56 ^b^	65.32 ^b^	70.36 ^a^
Filtek Z550	79.03 ± 2.27	64.96 ^a^	63.56 ^a^	63.56 ^a^	58.16 ^b^	66.66 ^a^	65.96 ^a^

Data are presented as mean ± SD (*n* = 5). Different superscript letters within the same composite indicate statistically significant differences between immersion media (one-way ANOVA with Tukey’s post hoc test, *p* < 0.05).

**Table 3:** Surface roughness Ra at baseline and after 28 days of immersion (µm, mean ± SD; *n* = 5).

Composite	Medium	Baseline Ra	Ra After 28 Days	ΔRa
Herculite	Water	0.162 ± 0.013	0.211 ± 0.037 ^a^	+0.049
	Tea		0.181 ± 0.011 ^ab^	+0.019
	Coffee		0.161 ± 0.019 ^b^	−0.001
	Coca-Cola^®^		0.161 ± 0.011 ^a^	−0.001
	Cola Light^®^		0.152 ± 0.008 ^c^	−0.010
	Red wine		0.164 ± 0.019 ^b^	+0.002
Herculite Ultra	Water	0.114 ± 0.023	0.128 ± 0.023 ^c^	+0.014
	Tea		0.123 ± 0.010 ^c^	+0.009
	Coffee		0.188 ± 0.024 ^a^	+0.074
	Coca-Cola^®^		0.150 ± 0.007 ^b^	+0.036
	Cola Light^®^		0.126 ± 0.016 ^c^	+0.012
	Red wine		0.125 ± 0.007 ^c^	+0.011
Filtek Z250	Water	0.121 ± 0.008	0.175 ± 0.023 ^b^	+0.054
	Tea		0.140 ± 0.021 ^c^	+0.019
	Coffee		0.173 ± 0.021 ^b^	+0.052
	Coca-Cola^®^		0.160 ± 0.023 ^b^^c^	+0.039
	Cola Light^®^		0.211 ± 0.054 ^a^	+0.090
	Red wine		0.193 ± 0.003 ^ab^	+0.072
Filtek Z550	Water	0.181 ± 0.027	0.159 ± 0.015 ^b^	−0.022
	Tea		0.173 ± 0.026 ^a^	−0.008
	Coffee		0.166 ± 0.007 ^ab^	−0.015
	Coca-Cola^®^		0.113 ± 0.010 ^c^	−0.068
	Cola Light^®^		0.146 ± 0.017 ^c^	−0.035
	Red wine		0.153 ± 0.037 ^b^	−0.028

ΔRa represents the difference between post-aging and baseline roughness values (ΔRa = Ra_after − Ra_baseline). Different superscript letters within the same composite indicate statistically significant differences between immersion media (one-way ANOVA with Tukey’s post hoc test, *p* < 0.05).

**Table 4:** Steady-state coefficient of friction (COF) determined in the stabilized sliding regime (final 30% of sliding distance).

Composite	Medium	COF (Mean ± SD)	ΔCOF
Herculite	Baseline	0.337 ± 0.036	-
	Water	0.393 ± 0.014	+0.056
	Tea	0.582 ± 0.031	+0.245
	Coffee	0.464 ± 0.002	+0.127
	Coca-Cola^®^	0.452 ± 0.036	+0.115
	Cola Light^®^	0.257 ± 0.064	−0.080
	Red wine	0.154 ± 0.019	−0.183
Herculite Ultra	Baseline	0.400 ± 0.024	-
	Water	0.297 ± 0.010	−0.103
	Tea	0.466 ± 0.012	+0.066
	Coffee	0.392 ± 0.010	−0.008
	Coca-Cola^®^	0.272 ± 0.032	−0.128
	Cola Light^®^	0.127 ± 0.011	−0.273
	Red wine	0.370 ± 0.020	−0.030
Filtek Z250	Baseline	0.541 ± 0.007	-
	Water	0.355 ± 0.073	−0.186
	Tea	0.601 ± 0.030	+0.060
	Coffee	0.481 ± 0.066	−0.060
	Coca-Cola^®^	0.606 ± 0.042	+0.065
	Cola Light^®^	0.522 ± 0.013	−0.019
	Red wine	0.490 ± 0.002	−0.051
Filtek Z550	Baseline	0.598 ± 0.069	-
	Water	0.468 ± 0.014	−0.130
	Tea	0.563 ± 0.061	−0.035
	Coffee	0.414 ± 0.035	−0.184
	Coca-Cola^®^	0.613 ± 0.006	+0.015
	Cola Light^®^	0.697 ± 0.060	+0.099
	Red wine	0.547 ± 0.018	−0.051

**Table 5:** Fluid sorption and mass variation in dental composite specimens after 28 days of aging (%).

Composite	Water	Tea	Coffee	Coca-Cola^®^	Cola Light^®^	Red Wine
Herculite	+3.751	+2.465	+4.234	+4.716	−1.715	+2.465
Herculite Ultra	+0.051	+1.516	−1.213	+0.606	+2.931	−0.707
Filtek Z250	+1.914	−0.048	−0.574	+0.622	+1.340	−2.440
Filtek Z550	+0.051	+0.509	+1.017	+1.628	+2.899	−0.203

## Data Availability

The original contributions presented in this study are included in the article. Further inquiries can be directed to the corresponding authors.
